# Genetic Polymorphisms and Their Impact on Body Composition and Performance of Brazilians in a 105 Km Mountain Ultramarathon

**DOI:** 10.3390/ejihpe13090127

**Published:** 2023-09-10

**Authors:** Marcelo Romanovitch Ribas, Fábio Kurt Schneider, Danieli Isabel Romanovitch Ribas, André Domingues Lass, Georgian Badicu, Júlio Cesar Bassan

**Affiliations:** 1 Postgraduate Program in Electrical Engineering and Industrial Informatics, Universidade Tecnológica Federal do Paraná, Curitiba 80230901, Brazil; mromanovitch@yahoo.com.br (M.R.R.); fabioks@utfpr.edu.br (F.K.S.); 2 Human Genetics Laboratory, Centro Universitário Autônomo do Brasil (UniBrasil), Curitiba 82821020, Brazil; danieliribas@yahoo.com.br; 3 Laboratory of Exercise Biochemistry in Health, Graduate Program in Health Sciences, School of Medicine, Pontifícia Universidade Católica do Paraná, Curitiba 80215901, Brazil; andrelass0974@gmail.com; 4 Department of Physical Education and Special Motricity, Faculty of Physical Education and Mountain Sports, Transilvania University of Braşov, 500068 Braşov, Romania; 5 Postgraduate Program in Physical Education, Universidade Tecnológica Federal do Paraná, Curitiba 81310900, Brazil; jcbassan@utfpr.edu.br

**Keywords:** polymorphism, genetic, athletic performance, marathon, running

## Abstract

Although the studied polymorphisms affect muscular proteins, aerobic adaptations, and recovery, their influence on the anthropometric variables and performance in ultramarathon runners is still poorly understood. This study aimed to determine the influence of *ACTN3 R577X*, *ACE I/D*, and *CK MM A/G NcoI* polymorphisms on the changes in the anthropometric variables and running time of 105 km mountain runners, in which 22 male Brazilian elite athletes (35.9 ± 6.5 years) were evaluated. Genotyping of the *R577X* (RR, RX, and XX), *ACE I/D* (DD, ID, and II), and *CK MM A/G Ncol* (AA, AG, and GG) polymorphisms was performed using the Polymerase Chain Reaction–Restriction Fragment Length Polymorphism (PCR-RFLP) technique with DNA extracted from saliva. Body composition was determined via bioimpedance. Pre- and post-race weight differences were observed on athletes with the AA genotype (77.1 ± 5.9 kg; 74.6 ± 5.6 kg) compared with those with the AG genotype (74.5 ± 8.0 kg; 68 ± 5.1 kg) (*p* = 0.02; *p* = 0.02). The RR genotype showed a correlation between BMI and running time (R = 0.97; *p* = 0.004). The genotype II showed a correlation with % fat and fat mass concerning running time (R = 0.91; *p* = 0.003; R = 0.99; *p* < 0.0001). The AA genotype was associated with post-race weight and lean mass loss, while the RR genotype correlated with BMI, and the genotype II correlated with % body fat and fat mass in relation to times in the 105 km mountain ultramarathon.

## 1. Introduction

Mountain ultramarathons are running events that encompass distances greater than the standard marathon length (42.195 km), featuring a significant cumulative elevation gain (up to 25,000 m) [1]. These races, despite requiring at least 10 h to complete and posing a strenuous physiological challenge to the human body [2], have witnessed a substantial increase in popularity over the past three decades [3]. This surge in popularity has spurred researchers to delve into the physiological adaptations associated with long-distance events [4].

Regarding predictor evaluation, behavioral, psychological, mechanical, and physiological factors, such as the maximal oxygen consumption and the lactate threshold, they may be linked to the final performance of long-distance runners [5,6,7]. Previous research has examined the association between anthropometric measures and the runners’ performance, revealing a correlation between a decrease in body mass index (BMI) [8] and body weight loss [9], with faster running times in ultramarathon athletes.

In another line of research, a study conducted by Belli et al. [10] demonstrated that weight loss among ultramarathon runners in 217 km races occurs within the first 84 km of the race and is then maintained until the race’s conclusion. Furthermore, this study concluded that higher accumulations of body fat, concentrated in the lower limbs and abdominal region, may have a negative impact on athletes’ performance in this type of event. Lower levels of body fat (≤12%) appear to provide advantages for a faster race [11].

However, despite evidence of a correlation between anthropometry and final performance, it is necessary to emphasize that genes and their polymorphisms are partially responsible for determining the physiological, anthropometric, and psychological characteristics needed to achieve athletic performance and elite athlete status [12]. Variables such as height and body mass index are highly heritable and both contribute to identifying talent in different sports [13]. In general, there are few studies that have considered the relationship between genes and their polymorphisms and body composition phenotypes in male or female athletes [14].

Nevertheless, there is substantial evidence that genes and their polymorphisms, such as the Alpha Actinin 3 gene (*ACTN3 R577X*), Angiotensin Converting Enzyme (*ACE I/D*), and Creatine Kinase MM Enzyme (*CK MM A/G Ncol*), influence muscle performance and metabolism in humans [15,16]. It is important to note that the ACTN3 R577X gene polymorphism is involved in the structure of fast-twitch muscle fibers. The presence or absence of the R577X polymorphism can influence the composition of muscle fibers, thereby affecting athletic performance [17]. Individuals with the XX genotype show higher percentages of Type I muscle fibers, a condition associated with higher volumetric densities of mitochondria, making them more resistant to muscle fatigue [18].

On the other hand, the ACE I/D polymorphism is associated with ACE activity, which plays a role in blood pressure regulation and the renin–angiotensin–aldosterone response mechanism [19]. The I allele of the ACE I/D polymorphism is associated with lower ACE levels, resulting in better endothelium-dependent vasodilation [20]. Individuals with the I allele have a 50% higher volumetric density of mitochondria and sarcoplasm, contributing to fatigue resistance [18]. On the other hand, the D allele is associated with higher ACE levels, which can influence cardiovascular function during exercise and oxidative stress responses [2].

Equally important, the CK MM A/G Ncol polymorphism is related to creatine kinase (CK), an enzyme involved in muscle energy production. Therefore, variations in this polymorphism can influence CK’s ability to regenerate after exercise, potentially affecting energy availability and muscle resilience during prolonged activities. This, in turn, can affect ATP availability for mitochondria and thus influence aerobic energy production in muscle cells [21].

It is worth emphasizing that the worldwide prevalence of genetic polymorphisms, including the *ACTN3 R577X* polymorphism, *ACE I/D*, and *CK MM A/G NcoI*, can vary among different athlete groups across various sports disciplines. Particularly for mountain ultramarathon athletes, these polymorphisms might manifest unique genotypic distributions, playing a pivotal role in adaptations and athletic performance. This is exemplified by studies covering power-oriented athletes [22], climbers [23], marathon runners [24], and ultramarathon runners [25].

However, upon searching the literature for the analyses of the association between body composition and genetics, only three studies have been documented, involving ballet dancers [26], rugby athletes [14], and Chinese rowing athletes [12]. This highlights the need for studies in the literature that explore the body composition of mountain ultramarathon runners, along with the analysis of the *ACTN3 R577X*, *ACE I/D*, and *CK MM A/G Ncol* gene polymorphisms. Filling this knowledge gap, this study aims to assess the influence of the *ACTN3 R577X*, *ACE I/D*, and *CK MM A/G NcoI* polymorphisms on changes in the anthropometric variables and race time in male mountain runners covering 105 km. The main hypothesis of this study posited that the *ACTN3 R577X*, *ACE I/D*, and *CK MM A/G NcoI* polymorphisms would exert an influence on the body composition variables and running time in mountain runners covering a distance of 105 km.

## 2. Materials and Methods

### 2.1. Subjects and Ethical Approval

The study was approved by the local Research Ethics Committee (CEP number 2.275.040). All study procedures were conducted in accordance with Resolution 466/12 of the Brazilian National Health Council and carried out during the Ultramaratona dos Perdidos SkyMarathon^®^, which took place at Morro dos Perdidos in Tijucas do Sul, Paraná. The racecourse ranged in altitude from 255 to 1654 m above sea level, including ascents of up to 1330 m and descents of up to 530 m. This study included 22 elite male athletes who competed in a 105 km mountain race, with ages ranging from 35.9 ± 6.5 years. The inclusion criteria established for athletes to participate in the study were as follows: having a minimum experience of at least two races above 50 km and one race above 80 km between 2015 and 2016; no reports of non-communicable chronic-degenerative diseases; and a cardiorespiratory examination for cardiovascular function assessment, certified by a cardiologist. Athletes who did not sign the Informed Consent Form (ICF), did not complete one of the two data collection stages, or expressed a desire to withdraw their consent were excluded from the study.

### 2.2. Anthropometric Assessment

The anthropometric evaluation consisted of measurements of body weight (BW), height, fat percentage (%F), fat mass (FM), and lean mass (LM). BW was measured on a platform-type anthropometric scale (Filizola, São Paulo, Brazil) with a precision of 100 g, and height was determined with a portable stadiometer (Seca, Hamburg, Germany) with a precision of 0.1 cm, considering the arithmetic mean of three consecutive measurements as the final value. From BW and height, the body mass index (BMI) was determined [27]. The body fat percentage (%F), fat mass (FM), and lean mass (LM) were determined using the bioimpedance tetra polar whole-body method (Maltron, Rayleigh, UK) with electrical frequencies of 50 kHz.

### 2.3. Genotyping

For all athlete samples, genomic DNA was extracted from saliva collected through scraping the buccal mucosa and rinsing with a 3% glucose solution, using a standard phenol–chloroform extraction method. All samples were stored in the freezer at −20 °C for two days [28]. For genotyping the ACTN3 R577X polymorphism, specific primers were used (Table 1). The PCR amplification protocol included (a) 95 °C for 5 min, (b) 94 °C for 30 s, (c) 58 °C for 30 s, (d) 72 °C for 30 s, (e) repeating steps “b” to “d” 30 times, and (f) 72 °C for 5 min. After amplification, the PCR product was subjected to digestion with the DdeI restriction enzyme (SIGMA Aldrich, St. Louis, MO, USA) at 37°C for 4 h [29].

For genotyping the ACE I/D polymorphism, specific primers were used (Table 1). The PCR amplification protocol included (a) 95 °C for 5 min of initial denaturation and enzyme release, (b) 30 cycles of denaturation at 94 °C for 30 s, (c) annealing at 57 °C for 1 min, (d) an extension at 72 °C for 1 min, and (e) a 5 min extension at 72 °C [28]. To enhance genotyping specificity, the samples showing the DD genotype were re-evaluated using an insertion-specific forward primer (Table 1). The PCR amplification protocol included (a) 95 °C for 5 min of initial denaturation and enzyme release, (b) 35 cycles of denaturation at 94 °C for 30 s, (c) annealing at 56 °C for 1 min, (d) an extension at 72 °C for 1 min, and (e) after finishing the 35 cycles, there was a 5 min extension at 72 °C [28].

For genotyping the CK MM A/G Ncol polymorphism, specific primers were used (Table 1). The PCR amplification protocol included (a) 1 cycle of denaturation at 95 °C for 5 min, (b) 35 cycles of denaturation at 95 °C for 30 s, (c) annealing at 66 °C for 30 s, (d) an extension at 72 °C for 1 min, and (e) a 10 min final elongation cycle at 72 °C. Following amplification, the PCR product was digested with the Ncol restriction enzyme (New England Biolabs, Beverly, MA, USA) at the specified conditions [30]. For genotype analysis, electrophoresis (Kasvi, São José do Pinhais, PR, Brazil) was performed in 3% agarose gel, stained with ethidium bromide, and visualized in a UV transilluminator.

### 2.4. Statistical Analysis

The data were analyzed using descriptive statistics (mean, standard deviation, and percentages) in R software, version 4.0.5. The Shapiro–Wilk test was used and showed that the pre- and post-race anthropometric variables (body weight, BMI, body fat percentage, fat mass, and lean mass) followed a normal distribution. Therefore, the influence of the *ACTN3 R577X*, *ACE I/D*, and *CK MM A/G NcoI* polymorphisms and their genotypes on the anthropometric variables at the pre- and post-race moments was verified using the Independent Samples *t*-test. Sample size calculation was performed using statistical power analysis software (GPower, Version 3.1.9.2, Aichach, Germany), adopting a standardized difference of 0.7, with α = 0.05 and power = 0.8 to be considered significant. Our power analysis suggested that finding a significant difference in measurement would require a total sample size of 19 subjects. The effect size (ES) magnitude between groups was calculated using Hedges’ g with a 95% confidence interval (CI). The correlation of the anthropometric variables (body weight, BMI, %F, FM, and LM) of the *ACTN3 R577X, ACE I/D*, and *CK MM A/G NcoI* polymorphisms and their genotypes with running time was evaluated using simple linear regression and categorized as follows: weak correlation (0.20–0.39), moderate correlation (0.40–0.59), strong correlation (0.60–0.79), and very strong correlation (0.80–1.0). For all statistical procedures, a significance level of *p* ≤ 0.05 was adopted. In order to minimize the Type I error, the Bonferroni correction was used, adjusting the significance level based on the number of tests conducted.

## 3. Results

Table 2 presents the results of the comparative analyses among different genotypes of the *ACTN3 R577X* polymorphism for each anthropometric variable, before and after the race. The results are organized according to the genotype comparisons: RR vs. RX, RX vs. XX, and RR vs. XX. Following the assessment of pre- and post-race mean values for the anthropometric variables with respect to the *ACTN3 R577X* polymorphism genotypes, no statistically significant differences were observed (*p* > 0.05).

Table 3 presents the results of the analyses comparing various genotypes of the ACE I/D and *CK MM A/G NcoI* polymorphisms for each anthropometric variable, both before and after the race. The genotypes are categorized as ID vs. II for *ACE I/D* and AA vs. AG, AA vs. GG, and GG vs. AG for *CK MM A/G NcoI*. After evaluating the mean values of the pre- and post-race anthropometric variables in relation to the genotypes of *ACE I/D* and *CK MM A/G NcoI* polymorphisms, the statistical analysis revealed that the *CK MM A/G NcoI* polymorphism (AA vs. AG) showed significant differences for weight and MM (*p* ≤ 0.05). This indicates that individuals with the AA genotype exhibited higher weight and lean mass compared to those with the AG genotype before and after the race. The remaining polymorphisms did not show significant differences for the statistical variables.

The results from Table 4 provide an overview of the simple linear regression analyses establishing the relationships between the anthropometric variables and the *ACTN3 R577X* genotype’s impact on running time in minutes for the study sample. Athletes with the RR genotype showed a strong correlation with BMI when compared to running time (R = 0.97; *p* = 0.004). The RX and XX genotypes did not exhibit a correlation between the anthropometric variables and running time (*p* > 0.05).

The results from Table 5 provide an overview of the simple linear regression analyses that assessed the relationships between the anthropometric variables of the *ACE I/D* genotype and running time for the study sample. The II genotype showed a significantly strong correlation with several body composition variables, including %F and FM, concerning running time. These findings suggest that this genotype may have a significant influence on the relationship between body composition and running time. The ID genotype showed no correlation between anthropometric variables when compared with running time (*p* > 0.05). The results of the DD genotype were not presented because only one athlete was genotyped with this genotype in the present study.

Table 6 summarizes the simple linear regression analyses between the anthropometric variables and the CK MM A/G NcoI genotype in relation to running time. The AA, AG, and GG genotypes showed no correlation between the anthropometric variables and running time (*p* > 0.05).

## 4. Discussion

To reiterate the aim of our study, we aimed to assess the influence of the *ACTN3 R577X*, *ACE I/D*, and *CK MM A/G NcoI* polymorphisms on changes in the anthropometric variables and running time in male mountain runners covering 105 km. The study hypothesis was partially addressed, as it was observed that not all genotypes of the *ACTN3 R577X*, *ACE I/D*, and *CK MM A/G NcoI* polymorphisms exhibited significant associations with the studied body composition variables and running time.

The results revealed that the AA genotype of the *CK MM A/G NcoI* polymorphism was associated with greater weight loss after the race. Furthermore, the relationship between the AA genotype and the loss of lean mass after the race provides intriguing insights into how genetics may play a role in the muscular response to prolonged exertion. The *CK MM* gene codes the creatine kinase, an enzyme involved in muscular energy production. Variations in this gene, such as the *CK MM A/G NcoI* polymorphism, can affect the enzyme’s ability to regenerate after exercise. This, in turn, can impact muscular energy availability during prolonged activities like long-distance running, influencing the body’s capacity to endure prolonged effort and post-exercise recovery [21].

Conversely, other anthropometric variables did not reveal statistically significant differences when analyzed according to the different genotypes of the studied polymorphisms. When correlating the anthropometric variables with running time, athletes with the RR genotype of the *ACTN3 R577X* polymorphism demonstrated a correlation with body mass index (BMI) in relation to running time. This connection highlights the interrelation between genetics, body composition, and performance in mountain running. The presence of the RR genotype of this polymorphism is associated with the full expression of the alpha-actinin-3 protein in fast-twitch muscle fibers, a crucial feature for long-duration and high-intensity activities like mountain ultramarathons [31]. The correlation with BMI may suggest that athletes with this genotype, who tend to have greater muscle mass, can more effectively handle the demands of mountain running, where muscular strength and endurance are vital [14,32].

Athletes with the II genotype of the *ACE I/D* polymorphism demonstrated correlations with the percentage of body fat (%F) and fat mass (FM) when compared with running time. However, the other genotypes did not exhibit correlations between body composition and running time. The I allele of the *ACE I/D* polymorphism is related to endurance disciplines and lower ACE levels, as well as a higher percentage of type I muscle fibers. This could favor a better balance between muscular endurance and body composition, contributing to a more effective performance in long-distance running [2].

It is understood that several factors can affect the performance and anthropometry in long-distance runners, although that depends on the distance covered, which is related to the final performance of the event [5]. Regarding body composition, when comparing pre–post-run body weight loss in the AA-GG genotype of the *CK MM A/G NcoI* polymorphism, the present study showed that the athletes with the AA-AG genotype lost (3.2–2.7%), respectively. Belli et al. [10] showed that after an ultramarathon of 217 km, the athletes lost 3.9% of their body weight after the end of the race. Martínez-Navarro et al. [5], when assessing weight loss in a 107 km mountain race, demonstrated that male athletes lost 4.37 ± 1.77% of their body weight by the end of the event. According to the same authors, greater weight losses could be expected among faster runners and during the faster segments in mountain ultramarathon races.

When comparing the pre–post-run lean mass in the AA-AG genotypes of the *CK MM A/G NcoI* polymorphism, the present study showed a loss of 3.7% in the athletes carrying the AA genotype and 0.32% in those with the AG genotype at the end of the race. Belli et al. [10] verified possible relationships between the decrease in lean mass and the performance of athletes in the 217 km ultramarathon, with a 3.04% loss being reported. Mueller et al. [33] reported that the loss of body weight after an Ironman Triathlon was due to a 4.5% loss in fat mass and a 2.4% loss in lean body mass, the latter being attributable to the loss of glycogen as fuel for the production of energy and the corresponding loss of body water.

Regarding the anthropometric variables, the *ACTN3 R577X* polymorphism, and running time, the RR genotypes showed a positive correlation with BMI, showing that athletes of the RR genotype had the lowest running times and lowest BMI values. The RR genotype is associated with the full expression of *ACTN3* in fast-type muscle fibers, which is highly prevalent among elite athletes in strength and power sports. The RR genotype may favor the ability to generate strong and vigorous muscle contractions, an important skill that increases running speed and, consequently, influences locomotion [17]. Furthermore, the RR genotype may also favor the ability to resist exercise-induced muscle damage [16].

In the present paper, the analysis of the anthropometric variables, the *ACE I/D* polymorphism, and running time showed that genotype II had a correlation between running time and %F and FM pre–post running, which presented a decrease of 10.5% and 14.1% in that order. Individuals with the II genotype exhibit a higher percentage of Type I fibers and may increase bradykinin levels and decrease *ACE* enzyme activity so the local concentration of nitric oxide in skeletal muscle increases, thus increasing the mitochondrial respiratory efficiency and the contractile function of skeletal muscle to improve human endurance performance and fat oxidation [34]. The I allele is associated with greater fat storage during physical training. Fat is an important source of energy for skeletal muscle, yet most elite distance runners have a low percentage of body fat mass. Heavier body mass consumes more energy with movement, so low-fat body mass is efficient for endurance running [35].

The role of the *CK MM A/G NcoI* gene in physical performance status has not been definitively established [36]. This present study is the first report on the *CK MM* gene’s *A/G NcoI* polymorphism in relation to the body composition of athletes and running time in a 105 km mountain ultramarathon. Concerning the anthropometric variables, the *CK MM A/G NcoI* genotype, and running time, athletes did not show a correlation between the anthropometric variables and running time. Fedotovskaia et al. [37] demonstrated that the A allele of *CK MM* influences gene expression, resulting in decreased activity of the muscle isoform of creatine kinase in myocytes, leading to increased oxidative phosphorylation and muscle endurance, a situation that may explain the findings in the present study. Athletes with the *CKMM GG* genotype, compared to those with the AA genotype, are six times less likely to exhibit an exaggerated CK response to exercise. Therefore, the G allele may be related to a protective mechanism against muscle breakdown due to exertion [37], which would partly explain the findings.

Despite the limitations, such as the absence of a control group and the lack of control over the food and supplement intake during the race, the results of this study suggest that the genetic polymorphisms *ACTN3 R577X*, *ACE I/D*, and *CK MM A/G NcoI* may play a complex role in the muscular response and body composition of mountain ultramarathon runners. The intersection of genetics and athletic performance underscores the importance of considering individual factors when assessing and training endurance athletes. For a more comprehensive understanding, it is recommended that future research explores these relationships on a larger and more comprehensive scale, including other demographic groups such as female runners, genetic and body composition comparisons between athletes who run at high altitudes and at sea level, and taking into account the waist circumference measurements of the athletes, in order to deepen our knowledge of how genes influence adaptability and performance in 105 km mountain ultramarathons. This information has the potential to inform more personalized and effective training strategies for athletes striving for excellence in challenging competitions, such as mountain ultramarathons.

## 5. Conclusions

Based on the results presented in this study, it is evident that genetic polymorphisms play a fundamental role in the muscular response, body composition, and athletic performance of mountain ultramarathon runners. Specific genotypes of the *ACTN3 R577X* and *ACE I/D* polymorphisms demonstrated significant correlations with the anthropometric variables and performance times in this type of competition. The *CK MM A/G NcoI* polymorphism’s AA genotype was associated with greater post-race weight and lean mass loss. On the other hand, the RR genotypes of the *ACTN3 R577X* polymorphism and the II genotype of the *ACE I/D* polymorphism exhibited correlations with body mass index (BMI) in relation to performance times in the 105 km mountain ultramarathon. Additionally, the II genotype of the *ACE I/D* showed associations with % fat and fat mass in relation to performance times. However, the genotypes of the *CK MM A/G NcoI* polymorphism did not show correlations between body composition and performance times. These conclusions underscore the importance of the complex interplay between genetics, body composition, and athletic performance in mountain ultramarathons. The findings suggest that specific genotypes of the studied polymorphisms influence the body’s responses to this strenuous type of competition.

## Figures and Tables

**Table 1:** Primers used and their respective gene accession numbers.

Gene	Primer Forward	Primer Reverse	Accession Number
*ACTN3*	5′-CTGTTGCCTGTGGTAAGTGGG-3′	5′-TGGTCACAGTATGCAGGAGGG-3′	89
*ACE*	5’-TGGGACCACAGCGCCCGCCACTAC-3’	5′-CTGGAGACCACTCCCATCCTTTCT-3′	1636
*CKMM*	5′-GTGCGGTGGACACAGCTGCCG-3′	5-CAGCTTGGTCAAAGACATTGAGG-3	1158

**Table 2:** Mean and standard deviation pre- and post-race of anthropometric variables related to the genotypes of polymorphism ACTN3R557X to the study sample (*n* = 22).

Polymorphism	Genotype Comparison							
*ACTN3 R577X*	**RX (*n* = 12) vs. RR (*n* = 5)**							
Variables	Pre	*p*-value	Hedges’ g	95% CI	Post	*p*-value	Hedges’ g	95% CI
Weight (Kg)	72.0 ± 6.1–70.7 ± 6.8	0.71	0.2	0.80–1.19	70.5 ± 5.4–69.5 ± 6.9	0.73	0.16	−0.83–1.15
BMI (Kg/m^2^)	24.5 ± 1.8–22.8 ± 2.6	0.18	0.79	−0.24–1.81	23.9 ± 1.5–22.4 ± 2.7	0.17	0.75	−0.27–1.77
%F	11.2 ± 2.7–9.9 ± 2.0	0.28	0.49	−0.52–1.49	9.6 ± 1.8–8.8 ± 1.9	0.42	0.42	−0.58–1.42
FM (Kg)	8.1 ± 1.7–6.9 ± 1.2	0.2	−0.84	−1.87–0.19	6.8 ± 1.5–6.0 ± 1.2	0.34	0.53	−0.47–1.54
LM (Kg)	64.0 ± 5.6–63.8 ± 6.9	0.93	0.05	−0.94–1.04	63.7 ± 4.6–63.4 ± 7.0	0.9	0.05	−0.94–1.04
	**RX (*n* = 12) vs. XX (*n* = 5)**							
Variables	Pre	*p*-value	Hedges’ g	95% CI	Post	*p*-value	Hedges’ g	95% CI
Weight (Kg)	72.0 ± 6.1–75.5 ± 7.8	0.33	−0.5	−1.51–0.50	70.5 ± 5.4–72.6 ± 7.6	0.53	−0.33	−1.33–0.67
BMI (Kg/m^2^)	24.5 ± 1.8–25.5 ± 2.7	0.38	−0.46	−1.46–0.55	23.9 ± 1.5–23.9 ± 1.1	0.94	0	−0.99–0.99
%F	11.2 ± 2.7–11.1 ± 2.5	0.95	0.04	−0.95–1.03	9.6 ± 1.8–8.8 ± 1.1	0.38	0.46	−0.54–1.46
FM (Kg)	8.1 ± 1.7–8.7 ± 2.4	0.57	−0.3	−1.29–0.70	6.8 ± 1.5–6.4 ± 2.2	0.58	0.22	−0.77–1.21
LM (Kg)	64.0 ± 5.6–68.8 ± 8.0	0.18	−0.72	−1.74–0.30	63.7 ± 4.6–66.2 ± 7.0	0.4	−0.44	−1.45–0.56
	**RR (*n* = 5) vs. XX (*n* = 5)**							
Variables	Pre	*p*-value	Hedges’ g	95% CI	Post	*p*-value	Hedges’ g	95% CI
Weight (Kg)	70.7 ± 6.8–75.5 ± 7.8	0.33	0.59	−0.56–1.74	69.5 ± 6.9–72.6 ± 7.6	0.53	0.39	0.75–1.52
BMI (Kg/m^2^)	22.8 ± 7.2–25.5 ± 2.7	0.16	0.32	−0.80–1.45	22.4 ± 2.7–23.9 ± 1.1	0.41	0.66	−0.50–1.81
%F	9.9 ± 2.0–11.1 ± 2.5	0.4	0.48	−0.66–1.62	8.8 ± 1.9–8.8 ± 1.1	0.85	0	−1.12–1.12
FM (Kg)	6.9 ± 1.2–8.7 ± 2.4	0.19	0.86	−0.32–2.04	6.0 ± 1.2–6.4 ± 2.2	0.75	0.2	−0.92–1.33
LM (Kg)	63.8 ± 6.9–68.8 ± 8.0	0.32	0.6	−0.55–1.76	63.4 ± 7.0–66.2 ± 7.0	0.46	−0.36	−1.49–0.77

*p* ≤ 0.05. BMI = body mass index; %F = body fat percentage; FM = fat mass; LM = lean mass.

**Table 3:** Mean and standard deviation pre- and post-race of anthropometric variables related to the genotypes of polymorphism *ACE I/D* e *CK MM A/G NcoI* to the study sample (*n* = 22).

Polymorphism	Genotype Comparison							
*ACE I/D*	**ID (*n* = 14) vs. II (*n* = 7)**							
Variables	Pre	*p*-value	Hedges’ g	95% CI	Post	*p*-value	Hedges’ g	95% CI
Weight (Kg)	71.6 ± 5.9–74.2 ± 7.5	0.37	−0.39	−1.27–0.49	69.6 ± 5.0–72.1± 7.7	0.37	−0.40	−1.28–0.48
BMI (Kg/m^2^)	24.5 ± 2.2–24.1 ± 2.6	0.68	0.16	−0.71–1.04	23.7 ± 1.9–22.9 ± 2.1	0.36	0.99	−0.49–1.27
%F	10.9 ± 2.7–10.4 ± 1.6	0.66	−0.24	−1.11–0.64	9.2 ± 1.5–9.3 ± 2.3	0.86	−0.05	−0.92–0.82
FM (Kg)	7.8 ± 2.1–7.8 ± 1.5	0.99	0.00	−0.87–0.87	6.4 ± 1.0–6.7 ± 2.0	0.57	−0.21	−1.08–0.67
LM (Kg)	63.9 ± 5.4–67.2 ± 8.2	0.26	−0.49	−1.38–0.39	63.2 ± 4.9–65.3 ± 6.7	0.43	−0.36	−1.24–0.51
**Polymorphism**	**Genotype Comparison**							
*CK MM A/G NcoI*	**AA (*n* = 7) vs. AG (*n* = 11)**							
Variables	Pre	*p*-value	Hedges’ g	95% CI	Post	*p*-value	Hedges’ g	95% CI
Weight (Kg)	77.1± 5.9–69.9 ± 5.7	0.02 *	1.19	0.20–2.17	74.6 ± 5.6–68 ± 5.1	0.02 *	1.19	0.21–2.17
BMI (Kg/m^2^)	25.6 ± 2.3–23.9 ± 2.0	0.13	0.76	−0.17–1.70	24.4 ± 2.0–23.3 ± 1.8	0.27	0.56	−0.36–1.48
%F	10.5 ± 1.8–11.2 ± 2.8	0.56	−0.27	−1.18–0.64	9.4 ± 2.4–9.1 ± 1.4	0.75	0.16	−0.75–1.06
FM (Kg)	8.2 ± 1.3–7.9 ± 2.4	0.79	0.14	−0.76–1.04	7.0 ± 1.8–6.2 ± 1.0	0.12	0.56	−0.36–1.48
LM (Kg)	70.3 ± 6.9–62.1 ± 4.2	0.006 *	1.45	0.43–2.47	67.7 ± 5.5–61.9 ± 4.8	0.03 *	1.09	0.12–2.06
	**AA (*n* = 7) vs. GG (*n* = 4)**							
Variables	Pre	*p*-value	Hedges’ g	95% CI	Post	*p*-value	Hedges’ g	95% CI
Weight (Kg)	77.1 ± 5.9–71.6 ± 6.8	0.19	0.81	−0.36–1.98	74.6 ± 5.6–70.9 ± 6.8	0.35	0.56	−0.59–1.71
BMI (Kg/m^2^)	25.6 ± 2.3–23.2 ± 2.4	0.14	0.94	−0.25–2.13	24.4 ± 2.0–23.0 ± 2.5	0.33	0.59	−0.56–1.74
%F	10.5 ± 1.8–10.6 ± 1.5	0.96	0.05	−1.07–1.18	9.4 ± 2.4–9.1 ± 1.3	0.78	0.13	−0.99–1.26
FM (Kg)	8.2 ± 1.3–7.5 ± 1.0	0.41	0.53	−0.62–1.67	7.0 ± 1.8–6.5 ± 1.4	0.61	0.27	−0.86–1.40
LM (Kg)	70.3 ± 6.9–64.0 ± 6.5	0.17	0.85	−0.33–2.03	67.7 ± 5.5–64.4 ± 5.6	0.37	0.55	−0.60–1.69
	**GG (*n* = 4) vs. AG (*n* = 11)**							
Variables	Pre	*p*-value	Hedges’ g	95% CI	Post	*p*-value	Hedges’ g	95% CI
Weight (Kg)	71.6 ± 6.8–69.9 ± 5.7	0.62	0.27	−0.81–1.35	70.9 ± 6.8–68.0 ± 5.1	0.42	0.49	−0.60–1.58
BMI (Kg/m^2^)	23.2 ± 2.4–23.9 ± 2.0	0.57	0.31	0.77–1.40	23.0 ± 2.5–23.3 ± 3.5	0.78	0.09	−0.99–1.16
%F	10.6 ± 1.5–11.2 ± 2.8	0.66	0.22	−0.86–1.30	9.1 ± 1.3–9.1 ± 1.4	0.92	0.00	−1.08–1.08
FM (Kg)	7.5 ± 1.0–7.9 ± 2.4	0.66	0.17	−0.90–1.25	6.5 ± 1.4–6.2 ± 1.2	0.74	0.23	−0.85–1.31
LM (Kg)	64.0 ± 6.5–62.1 ± 4.2	0.50	0.37	−0.71–1.46	64.4 ± 5.6–61.9 ± 4.8	0.41	0.47	−0.62–1.56

* *p* ≤ 0.05. BMI = body mass index; %F = body fat percentage; FM = fat mass; LM = lean mass.

**Table 4:** Simple linear regressions of anthropometric variables of the *ACTN3 R577X* genotype and running time for the study sample (*n* = 22).

Genotype	Variables	R	R^2^	Adjusted R	Std. Error	*p*-Value	Adjusted *p*-Value
**RR (*n* = 5)**	Weight (Kg)	0.56	0.31	0.08	9.4	0.32	1.6
	BMI (Kg/m^2^)	0.97	0.95	0.94	6.1	0.004	0.004 *
	%F	0.20	0.04	−0.27	40.7	0.74	3.7
	FM (Kg)	0.54	0.29	0.06	55.2	0.34	1.7
	LM (Kg)	0.46	0.21	−0.04	10.6	0.43	2.15
**RX (*n* = 12)**	Weight (Kg)	0.32	0.10	0.01	9.4	0.29	3.48
	BMI (Kg/m^2^)	0.03	0.00	−0.09	64.5	0.90	10.8
	%F	0.18	0.03	−0.06	28.4	0.55	6.6
	FM (Kg)	0.24	0.06	−0.03	33.2	0.44	5.28
	LM (Kg)	0.30	0.09	0.00	11.2	0.34	4.08
**XX (*n* = 5)**	Weight (Kg)	0.63	0.40	0.20	10.2	0.25	1.25
	BMI (Kg/m^2^)	0.83	0.69	0.59	25.3	0.07	0.35
	%F	0.07	0.00	−0.32	87.8	0.90	4.5
	FM (Kg)	0.30	0.09	−0.21	87.0	0.61	3.05
	LM (Kg)	0.63	0.40	0.21	11.1	0.24	1.2

* *p* ≤ 0.05. BMI = body mass index; %F = body fat percentage; FM = fat mass; LM = lean mass.

**Table 5:** Simple linear regression between anthropometric variables of the *ACE I/D* genotype and running time for the study sample (*n* = 22).

Genotype	Variables	R	R^2^	Adjusted R	Std. Error	*p*-Value	Adjusted *p*-Value
**ID (*n* = 14)**	Weight (Kg)	0.47	0.22	0.15	9.6	0.08	1.12
	BMI (Kg/m^2^)	0.44	0.19	0.12	25.4	0.11	1.54
	%F	0.24	0.06	−0.01	35.3	0.38	5.32
	FM (Kg)	0.47	0.22	0.15	46.5	0.08	1.12
	LM (Kg)	0.38	0.14	0.07	10.2	0.17	2.38
**II (*n* = 7)**	Weight (Kg)	0.63	0.40	0.28	6.28	0.12	0.84
	BMI (Kg/m^2^)	0.82	0.67	0.61	17.02	0.02	0.14
	%F	0.91	0.84	0.81	10.7	0.003	0.003 *
	FM (Kg)	0.99	0.99	0.98	3.08	<0.0001	<0.0001 *
	LM (Kg)	0.43	0.18	0.02	8.46	0.33	2.31

* *p* ≤ 0.05. BMI = body mass index; %F = body fat percentage; FM = fat mass; LM = lean mass.

**Table 6:** Simple linear regression between anthropometric variables of the *CK MM A/G NcoI* genotype and running time for the study sample (*n* = 22).

Genotype	Variables	R	R^2^	Adjusted R	Std. Error	*p*-Value	Adjusted *p*-Value
**AA (*n* = 7)**	Weight (Kg)	0.09	0.00	−0.18	10.5	0.83	5.81
	BMI (Kg/m^2^)	0.63	0.39	0.27	22.9	0.12	0.84
	%F	0.15	0.02	−0.17	24.2	0.35	2.45
	FM (Kg)	0.15	0.02	−0.17	32.5	0.73	5.11
	LM (Kg)	0.15	0.02	−0.17	10.5	0.74	5.18
**AG (*n* = 11)**	Weight (Kg)	0.68	0.46	0.40	8.9	0.02	0.22
	BMI (Kg/m^2^)	0.58	0.34	0.27	27.1	0.05	0.55
	%F	0.08	0.00	−0.10	43.0	0.80	8.8
	FM (Kg)	0.21	0.04	−0.05	56.5	0.52	5.72
	LM (Kg)	0.67	0.45	0.39	9.5	0.02	0.22
**GG (*n* = 4)**	Weight (Kg)	0.90	0.82	0.73	9.5	0.09	0.36
	BMI (Kg/m^2^)	0.72	0.52	0.28	42.2	0.27	1.08
	%F	0.68	0.47	0.21	82.6	0.31	1.24
	FM (Kg)	0.85	0.72	0.59	54.6	0.14	0.56
	LM (Kg)	0.88	0.78	0.67	12.8	0.11	0.44

*p* ≤ 0.05. BMI = body mass index; %F = body fat percentage; FM = fat mass; LM = lean mass.

## Data Availability

Data are not publicly available due to privacy restrictions.
